# Trial by trial decoding of decisions in monkey MT cortex from small neuronal populations

**DOI:** 10.1186/1471-2202-14-S1-P280

**Published:** 2013-07-08

**Authors:** Ramon Nogueira, Jan Drugowitsch, Jordi Navarra, Rubén Moreno-Bote

**Affiliations:** 1 Research Unit, Parc Sanitari Sant Joan de Deu and Universitat de Barcelona, Esplugues de Llobregat, Barcelona, 08950, Spain; 2 Centro de Investigación Biomédica en Red de Salud Mental (CIBERSAM), Esplugues de Llobregat, Barcelona, 08950, Spain; 3 Institut National de la Santé et de la Recherche Médicale & École Normale Supérieure, Paris, 75005, France

## Introduction

Classical experiments have shown that sensory neurons carry information about ongoing decisions during random-dot motion discrimination tasks [1,2]. These conclusions are based on the receiver-operating-characteristic (ROC) applied to single-cell recordings, which assumes the presence of a second hypothetical "anti-neuron". Furthermore, ROC analysis is by definition a measure of the average correlation between neuronal activity and stimulus/decisions. These limitations make ROC analysis unsuitable to study information about stimuli and decisions in neuronal populations on a trial by trial basis. In this study, using Poisson-like decoders, we inferred the information that a pair of motion selective MT neurons carries about the direction of motion of a random-dot stimulus presented to a monkey and about the ongoing formation of the decision of the animal. We found that Poisson-like decoders outperformed ROC analysis in both predicting stimulus and decision, with the predictive power of the Poisson-like decoders being twice that of the ROC analysis when decoding decisions. Because our framework is fully Bayesian, we could also detect signals that correspond to the belief that the animals have about their decisions, and track the time evolution of those signals. Our theory explains well-known behaviors in the meta-cognition literature such as underconfidence in easy trials, and sets a basis to study decision confidence in decision making using multielectrode recordings.

## Results

**Figure 1:**
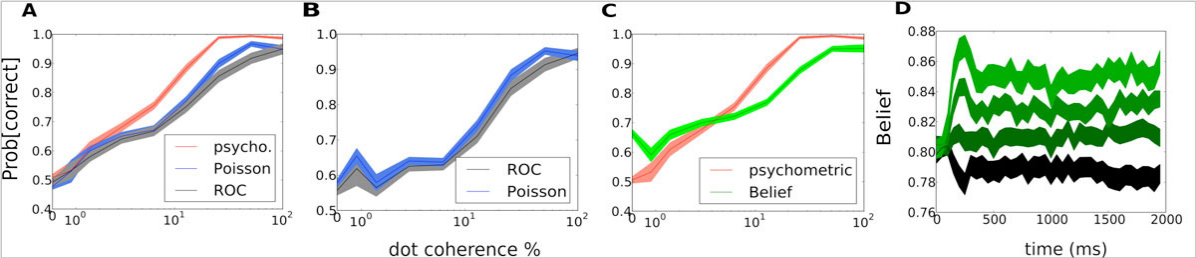
**A,B** Stimulus and decision decoding as a function of coherence. **C **Belief and performance as a function of coherence. Overconfidence for difficult trials and underconfidence for easy trials. **D **Degree of belief as a function of time for a sample of representative dot coherence.

Poisson-like decoders improve the results obtained by ROC in both stimulus and decision decoding (Figure 1A, B). In stimulus decoding (Figure 1A), at high coherences, the Poisson-like decoders outperform ROC. In decision decoding (Figure 1B), the Poisson-like decoders always beat ROC analysis. At zero coherence, ROC performance corresponds to Choice Probability (CP). The Poisson-like decoder nearly doubles ROC at zero coherence when compared to chance performance. Furthermore, our model provides an explanation to the overconfidence in difficult trials and underconfidence for easy trials by comparing the belief with the monkey performance (Figure 1C). The degree of belief during the latency period is determined by the previous trial and afterwards is modulated to the to the specific trial's degree of belief (Figure 1D). Belief's time modulation is different for each coherence value (different colors in Figure 1D). Belief in Figure 1C is evaluated for 2000 ms time bin, in Figure 1D is used a 50 ms time bin.

## Methods

Assuming an independent Poisson (PI) firing process, the probability of preferred direction and null direction stimulus given the monkey's decision and spike count was computed. Parameters were learned using maximum likelihood techniques from a training set and results shown correspond 10-fold cross-validated performance.
